# Research on the Formation Mechanism of Individual Food Waste Behavior from the Perspective of Image Construction

**DOI:** 10.3390/foods11091290

**Published:** 2022-04-29

**Authors:** Feiyu Chen, Xiao Gu, Jing Hou

**Affiliations:** 1 School of Economics and Management, China University of Mining and Technology, No. 1 Daxue Road, Xuzhou 221116, China; chenfeiyu@cumt.edu.cn (F.C.); ts21070116a31@cumt.edu.cn (X.G.); 2 Business School, Jiangsu Normal University, No. 101 Shanghai Road, Xuzhou 221116, China

**Keywords:** appearance image, social image, food waste behavior, formation mechanism

## Abstract

The attention regarding individuals’ external appearance and social identity provides a unique perspective to reveal the cause of their behavior. This study explored the formation mechanism of individual food waste behavior in China from the perspective of appearance image construction and social image construction, especially considering the role of emotion, education level, and body mass index (BMI) in relationship transmission. This study collected data by questionnaire in 133 cities in 32 provinces of China. By using the methods of factor analysis, correlation analysis, and hierarchical regression analysis, the results show that individuals with high need for external appearance image (r = 0.242, *p* < 0.001) and social image construction (r = 0.31, *p* < 0.001) are more likely to waste food. In terms of transmission mechanisms, positive emotions (e.g., excitement) (β = 0.104~0.187, 95% confidence interval) and negative emotions (e.g., anxiety and disgust) (β = 0.08~0.177, 95% confidence interval) are the intermediary factors of image construction affecting food waste behavior, and emotional fluctuations can aggravate individuals’ food waste behavior. In terms of interaction effects, BMI significantly positively regulates the predictive effect of image construction on food waste behavior, while the level of education buffers this predictive effect. Finally, relevant policy suggestions are put forward to guide individuals to reduce food waste.

## 1. Introduction

According to the report made by the Institute of Science and Technology Strategic Consulting of the Chinese Academy of Sciences in 2020, China’s annual food waste is approximately 67.5 million kg [1]. Food waste means the ineffective loss of human and material resources invested in the production of these foods [2], and it also endangers the country’s resource and environmental security [3], which has an important impact on global sustainable development [4]. Notably, food waste is different from food loss. Food loss refers to the reduction in the quantity or quality of edible food in the process of production, harvest, processing, and transportation [5]. Food waste generally refers to any raw or cooked quality food along the value chain which is suitable for human consumption but has not been unconsumed or discarded, and it is usually at the retail or consumer end of the chain [6]. In reality, more than 50% of food waste occurs in the final consumption stage; that is, the downstream consumption side of the supply chain is the main source of food waste [7]. Therefore, how to guide individuals to reduce food waste and raise the awareness of resource conservation is an important issue that needs to be solved for sustainable development at this stage.

Individual behavior motivations may have a significant impact on behavioral decision-making [8]. Similarly, motivation has an impact on food waste behavior. As we all know, needs are the root of motivation [9], and the positive evaluation of individual appearance by the outside world is an important need of individuals at this stage [10]. In the research regarding food waste behavior, Joyce et al. [11] found that individuals’ excessive attention to appearance image may easily lead to physical intention disorder, which in turn may affect their food waste behavior. Furthermore, social identity theory suggests that the need for individuals to be recognized by groups will also promote them to produce specific behavior [12]. Rachel [13] showed that in the process of social interaction, in order to obtain social identity and create a good social image, individuals will be extravagant and wasteful when inviting guests to dinner. Based on this, our study incorporates individuals’ external image construction needs and social image construction needs into a unified need construction framework, and explores the mechanism of image construction on individuals’ food waste behavior.

The generation of individual behavior is a complex process, and different factors will conduct or interact in the process of transforming needs into behavior [14]. Among them, the emotional attribution theory holds that when objective things or situations stimulate the needs of an individual, it will cause corresponding positive or negative emotions [15]. Moreover, emotions can inspire an individual’s behavior and become a powerful motivator to drive the individual’s behavior [16]. It can be seen that emotions may be the link between image construction needs and food waste response behavior. However, in view of the heterogeneity of individuals [17], although image construction needs and emotions can affect individual food waste behavior, this influence will be different due to individual heterogeneity. Studies have shown that with the improvement of individual education, individual food waste behavior tends to decrease [18]. In addition, studies have found that individuals with high body mass index (BMI) are significantly more dissatisfied with physical obesity than those with low BMI, and tend to waste more food during meals [19]. Based on this, an in-depth investigation of the mechanism of individual emotions, education level, and BMI in the process of transforming image construction needs into food waste behavior is helpful to grasp its formation law.

Therefore, this study aims to explore the formation mechanism of individual food waste behavior in China from the perspective of appearance image construction and social image construction, especially considering the role of emotion, education level, and BMI in relationship transmission. The relevant research conclusions are expected to provide support for the government to formulate intervention policies to reduce individual food waste behavior. The rest of the paper is structured as follows. Section 2 introduces the theoretical framework and research hypotheses of the paper. Section 3 describes the research methodology of this paper. Section 4 presents the empirical analysis of this research and Section 5 discusses the results of this research. Section 6 draws the conclusions of this research and makes corresponding policy recommendations.

## 2. Literature Review and Hypotheses

### 2.1. Concept and Dimensions of Food Waste Behavior

Food waste refers to the part of food that humans purchase but do not eat (i.e., discard) [4,20,21]. From the existing literature, scholars have different focuses and research directions for food waste behavior. For example, Cheng et al. [22] classified food waste in terms of where it occurs, and conducted in-depth research on household food waste and restaurant food waste, respectively; whereas Miranda et al. [23] classified food waste in terms of the types of food wasted. In addition, Pocol et al. [24] categorized consumers into three groups (i.e., careless, precautious, and ignorant) and studied the differences of these three groups in food waste behavior. However, in the model of food waste formation pathways, individual motivations and psychological mechanisms are usually ignored. Therefore, this paper focuses on food waste at the downstream consumption end of the supply chain and brings motivation and consciousness into the research scope, classifying food waste behavior as exclusion type, disregard type, extravagant type, and interference type. The food waste caused by the individual’s dislike of food or rejection of eating is called exclusion-type waste behavior; the waste caused by individuals disregarding the importance of food and the harm of waste to the environment is called disregard waste behavior; the pursuit of excessive enjoyment and extravagance when ordering is called extravagant waste behavior; and the food waste caused by the negative emotion of individuals when they are disturbed by the outside world is called interference waste behavior.

### 2.2. Predictive Effect of Image Construction Factors on Food Waste Behavior

The theory of planned behavior points out that individuals’ behavior is affected by their behavioral motivation, and individuals’ behavioral motivation depends on their attitude and subjective norms [25,26]. McComb and Mills [27] found that dissatisfaction with appearance is the direct motivation for eating disorders and food waste. Appearance discrimination is a common occurrence in everyday life, and the mainstream culture of society is that thinness is beautiful [28]. Individuals tend to improve their appearance image with the help of dietary adjustment [29]. Studies have also shown that individuals with appearance anxiety are more likely to suffer from anorexia nervosa and thus tend to waste food [30].

Individual behavior is the result of complex decision-making, and predicting food waste behavior needs to comprehensively consider many factors [31,32]. Image construction that reflects self-awareness will affect food waste behavior; in addition, subjective norms will also exert social pressure on individuals [33]. Mayo proposed the “social person hypothesis” [34], which states that the social environment acts on the mental side of the person and has a significant impact on individual behavior. What individuals show in the social environment and forms others’ cognition of individuals is their social image [35]. In order to obtain more social support, individuals tend to create a good social image [36] and take the amount of wasted food as the value measure to judge their interpersonal relationships, which will obviously cause serious food waste.

In addition, social learning theory holds that individuals’ perception of self-efficacy is an important antecedent factor that affects behavior [37]. The result of the behavior can stimulate and maintain the motivation of the behavior. The individual obtains the perceived benefits through the food waste behavior, which will directly affect the behavior tendency and stimulate the production of food waste behavior. Therefore, this study puts forward the following hypothesis.

**H1.**  *The factors of image construction have a significant positive effect on food waste behavior*.

More specifically, we can refine the above hypothesis as follows.

**H1a.**  *Appearance image construction significantly and positively influences individuals’ willingness to waste food*.

**H1b.**  *Social image construction significantly and positively influences individuals’ willingness to waste food*.

### 2.3. Mediating Role of Emotions

The cognitive theory of emotion asserts that emotions arise from the evaluation of stimulus situations [38,39]. The generation of emotions is influenced by three factors, i.e., environmental events, physiological conditions, and cognitive processes. Rachlin [40] pointed out that cognition and emotion can positively predict behavior. Similarly, the individuals’ perceptions and emotions of image construction will affect the individuals’ food waste behavior. Research has found that the emotions that arise during the process of shaping the image of appearance are complex. Individuals will produce positive emotions such as pleasure and expectation due to their longing for a beautiful appearance, and tend to reduce the negative emotions of appearance anxiety [41]. However, individuals will also have negative emotions such as shame and depression due to lack of self-control and difficulty in persisting in shaping their appearance image for a long time [42]. From the perspective of social image construction, although a good social image can enable individuals to obtain positive emotions such as pride, some people have social anxiety disorders when they are in a social situation and tend to show negative emotions such as fear [43].

Emotional variables are important factors that affect individual food waste behavior. The Antecedent-Belief-Consequence (ABC) theory of emotion points out that individuals with different emotions will have different behavior patterns when facing the same thing [44]. Positive emotions can have an effect on the individual’s self-regulation function (e.g., bring about an impulsive consumption tendency) and have a greater chance of causing food waste behavior [45]. Studies have also shown that negative emotions increase the probability of food waste [46]. Macht [47] found that 39% of the research results showed that negative emotions reduce eating. Although previous research regarding emotional variables has been carried out, the specific mechanism of emotion needs to be further explored. In summary, this study proposes the following hypothesis.

**H2.**  *Emotions play an intermediary role in the process of image construction affecting food waste behavior*.

### 2.4. Moderating Effect of Education Level and BMI

There is heterogeneity among individuals, which will interfere with individual behavior [17]. Studies have shown that education level affects the process of emotion processing and regulation, and individuals with low education level are vulnerable to emotional manipulation [48]. Individuals with high educational level receive more education, have strong self-control and more awareness of environmental protection, and have constraints on their own behavior [49]. Research has generally concluded that both low and high BMI can have an impact on emotion [50]. The World Health Organization has made BMI the World Health Organization’s screening standard for measuring overweight and obesity [51]. Women with low BMI believe that their appearance image has high attractiveness and thus have positive emotions about their appearance [52]. People with a high BMI index are prone to negative emotions such as depression and anxiety, and even have eating disorders [19]. From the perspective of behavioral motivation, individuals with a high BMI have a stronger motivation to lose weight and are more likely to engage in food waste behavior [53].

Based on this, the study introduces education level and BMI to explore the moderating role of education level and BMI in the mechanism of the effect of image construction on food waste behavior. The study proposes the following hypotheses.

**H3.**  *The mediating process by which image construction influences food waste behavior through emotions is influenced by the level of individual education*.

**H4.**  *The mediating process by which image construction influences food waste behavior through emotion is influenced by the individual’s BMI.*

**H5.**  *The direct predictive effect of image construction on food waste behavior is moderated by the level of individual education.*

**H6.**  *The direct predictive effect of image construction on food waste behavior is moderated by the individual’s BMI.*

**Figure 1:**
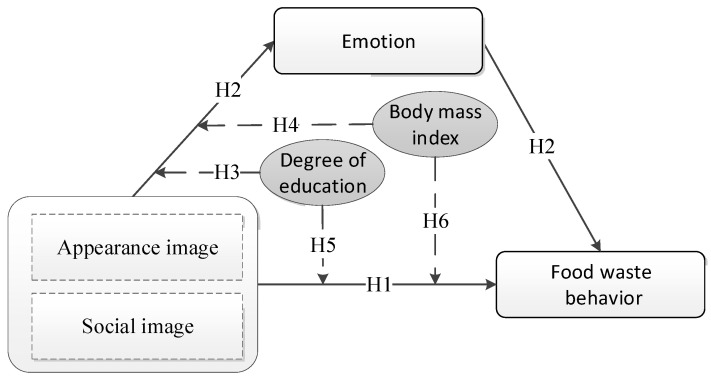
Conceptual model of food waste behavior.

In summary, this study constructs a conceptual model that explores the mechanisms of the role of image construction on individual food waste behavior (see Figure 1).

## 3. Methods

### 3.1. Scale Design

Based on the existing mature scales related to image construction, emotion, and food waste behavior [54,55,56,57], this study modified the items of the scale to form a comprehensive measurement scale combining the specific manifestations of image construction, emotion, and behavior. All items are measured by Likert 5, where “1” represents “not conformed” with the real situation, “2” represents “little conformed”, “3” represents “common”, “4” represents “conformed”, and “5” represents “quite conformed”.

The final research questionnaire is divided into four parts: The first part is a survey of basic individual information, mainly including the sample’s gender, age, education level, height, weight, income, and number of household members; the second part is the image construction scale, which is used to measure the level of image construction needs of the subjects; the third part is the emotion scale; and the fourth part is the food waste behavior scale, which is used to measure the types and motivation of subjects’ food waste behavior.

### 3.2. Research Sample and Data Collection

A stratified sampling method was used to determine the sample structure of the research so that the sample was distributed across a reasonable range of gender, age, income, and education levels. Particularly, to avoid being influenced by the differences in China’s population distribution and economic structure, this study chose to distribute the questionnaire on a large scale nationwide. The questionnaire was distributed from 13 January 2021 to 25 July 2021. In order to make up for the inconvenience of field investigation caused by the epidemic situation, the sample size was expanded through online investigation. The questionnaire link and QR code were generated through the domestic professional questionnaire platform, and then the questionnaire was delivered through various channels such as social networking sites, search engines, and shopping platforms. Additionally, internships or parent work groups were used to distribute questionnaires to various social residents. We issued a certain amount of red-envelopes to stimulate the participants’ willingness to fill in the questionnaires. The sample surveyed was spread over 133 cities in 32 provinces across the country. A total of 885 questionnaires were distributed in this study. Among them, 136 invalid questionnaires that were obviously answered randomly were removed. The final number of valid sample questionnaires was 749, and the effective rate of the questionnaire was 84.6%.

Among the surveyed samples, males accounted for 38.9% and females accounted for 61.1%. Samples under 17 years old accounted for 1.1%, samples aged 18–25 accounted for 47.5%, samples aged 26–30 accounted for 8.3%, samples aged 31–40 accounted for 11.2%, samples aged 41–50 accounted for 17.8%, samples aged 51–60 accounted for 10.3%, and samples over 60 years old accounted for 3.9%. Samples with high school or below accounted for 28%; samples with a college degree accounted for 8.4%, samples with a bachelor degree accounted for 53.9%, samples with master’s education accounted for 8.7%, and samples with doctoral education accounted for 0.9%. The proportion of samples with too light BMI was 15.6%, the proportion of samples with normal BMI was 59.9%, the proportion of samples with overweight BMI was 19.9%, and the proportion of samples with obese BMI was 4.5%. Overall, the sample is reasonable. The demographic characteristics of the sample are shown in Table 1.

According to the research methods of Lian [58] and Luo [59], income, gender, marriage, and age were put into the first layer as covariates in the subsequent hierarchical regression analysis.

### 3.3. Reliability and Validity Analysis

The reliability and validity of the questionnaire were tested, and the results show that the reliability and validity of the questionnaire were at a good level. The Cronbach’s coefficients for the food waste behavior and its related scales were all above 0.8 and the correlation coefficients between the items and the overall were all above 0.75. The results of Bartlett’s sphericity test showed that the KMO value of each scale was above 0.78 and the significance level of Bartlett’s sphericity test was less than 0.001, indicating that the effectiveness of all scales passed the preliminary test and was suitable for factor analysis. Except for food waste behavior, all other variables in this study were unidimensional and were not subjected to factor analysis.

The KMO value of the food waste behavior scale was 0.915 and the significance of Bartlett’s spherical test value was less than 0.001, indicating that the data were suitable for factor analysis. In this study, exploratory factor analysis of the food waste behavior scale was implemented with SPSS 22.0. The food waste behavior scale was used as the dependent variable, and principal component analysis was adopted to extract four common factors from the food waste behavior scale (see Table 2). The Cronbach values of the four types of food waste were 0.874, 0.782, 0.888, and 0.844, respectively. It can be seen from Table 2 that the question items are well distributed on four common factors, and the Cronbach’s coefficient for each construct in this present model were all greater than 0.6 [60], indicating high internal consistency and stability in this study. Thus, food waste behavior can be divided into four types, i.e., exclusion food waste behavior, disregard food waste behavior, extravagant food waste behavior, and interference food waste behavior.

## 4. Data Analysis and Hypothesis Testing

### 4.1. Descriptive and Correlation Analysis

The data were analyzed by descriptive and correlation analysis (see Table 3). From Table 3, we observed that the appearance image construction was significantly positively correlated with food waste behavior, and the correlation coefficient values was 0.242; social image construction was significantly positively correlated with food waste behavior, and the correlation coefficient values was 0.31; and they were significant at the level of 0.001. Therefore, hypothesis H1 was preliminarily verified.

Appearance image construction was significantly positively correlated with positive emotion and negative emotion; the correlation coefficients were 0.308 and 0.176, respectively, and they were significant at the level of 0.001. Social image construction was significantly positively correlated with positive emotion and negative emotion; the correlation coefficients were 0.387 and 0.239, respectively, and they were significant at the level of 0.001. We observed that there was a significant positive correlation between emotion and food waste behavior. It can be found that image construction can explain emotions and emotions can explain food waste behavior. Additionally, the critical value of the correlation level in Table 3 basically did not exceed 0.7, indicating that there was no serious multicollinearity problem in the data used.

### 4.2. Analysis of Impact Mechanism

#### 4.2.1. Verification of the Impact of Image Construction on Food Waste Behavior

In order to explore whether image construction promotes individual food waste behavior, a hierarchical regression analysis was conducted with food waste behavior as the dependent variable. Control variables (income, age, gender, and marriage), appearance image, and social image were introduced as independent variables in turn. The results are shown in Table 4.

According to the regression results in Table 4, after adding the image construction variable to the model, the explanatory variation of the regression equation increases to 13.7%, 6.2%, 11.8%, and 11.1%, respectively. This indicates that the explanatory effect of the regression equation is improved. Therefore, image construction has a significant positive impact on food waste behavior and hypothesis H1 is verified. Further research showed that the predictive effect of social image construction was stronger than that of appearance image construction.

#### 4.2.2. Verification of Mediation

Regression analysis was carried out using Model 4 of the SPSS Process component, and the bootstrap method was selected for the confidence interval estimation test. The sampling was repeated 5000 times, and the 95% confidence interval was calculated. For the convenience of analysis, the appearance image construction and social image construction were recorded as X1 and X2, respectively, the negative emotion and positive emotion were recorded as M1 and M2, respectively, and the four types of food waste behavior were recorded as Y1, Y2, Y3, and Y4, respectively.

First, taking the appearance image construction as the independent variable and four types of food waste behavior as the dependent variable, this study discussed the mediating role of emotion. The results are shown in Table A1 (in Appendix A). We observed that the confidence interval did not contain 0, and both the positive emotions and negative emotions actively mediate the influence of appearance image construction on individual food waste behavior willingness. Taking the path of “appearance image construction → exclusion food waste behavior” as an example, the partial mediator equation model for emotion is established. Part of the mediating effect of negative emotion was 0.08, and the part of mediating effect of positive emotion was 0.126. The mediating effect of positive emotion is stronger. This is also the case of “appearance image construction → interference food waste behavior”. For the two paths of “appearance image construction → disregard food waste behavior” and “appearance image construction → extravagant food waste behavior”, the mediating effect of negative emotions is stronger than that of positive emotions.

Next, taking social image construction as the independent variable and four types of food waste behavior as dependent variables, this study further explored the mediating role of emotions. The results are shown in Table A2 (in Appendix A). We observed that positive emotions had a stronger mediating effect in the two paths of “social image construction → exclusion food waste behavior” and “social image construction → interference food waste behavior”. In the other two paths, the mediating effect of negative emotion is stronger than that of positive emotion.

From Table A1 and Table A2, we observed that both the positive emotion and negative emotion played a mediating role. Take the path of “appearance image construction → food waste behavior” as an example. When individuals reduce eating to shape their appearance image, the resulting emotions are different due to individual differences. Individuals with strong food dependence will have negative emotions due to reduced eating, which will reduce the interest in eating and increase food waste. When individuals have high appearance image construction needs, it will lead to a high level of food waste under the stimulation of positive emotions. This is also the case of “social image construction → food waste behavior”. Therefore, hypothesis H2 is verified.

#### 4.2.3. Verification of Regulation

This study used the PROCESS plug-in of SPSS 22.0 to test for moderating effects with reference to the bootstrap method proposed by Hayes [61]. The Model 8 of the SPSS Process component was used for regression analysis, sampling was repeated 5000 times, and a 95% confidence interval was calculated. Appearance image construction and social image construction are recorded as X1 and X2, respectively, the negative emotion and positive emotion are recorded as M1 and M2, respectively, the four types of food waste behavior are recorded as Y1, Y2, Y3, and Y4, respectively, and the adjustment variables educational level and BMI are recorded as W1 and W2, respectively.

First, income, age, gender, and marriage were taken as covariates to test the regulatory role of education level in the intermediary process of image construction affecting food waste behavior through emotion. The results are shown in Table A3 (in Appendix A). The results of data analysis show that education level can adjust the mediation model. When the education level is positive by one standard deviation, the 95% bootstrap confidence interval contains 0, and the mediation effect is not significant; however, the education level has a significant moderating effect in other cases. When the educational level is different, the size of the mediation effect will also be different; that is, when the educational level is low, the positive impact of image construction on individual food waste behavior through emotion is more significant, and such positive impact gradually weakens with the improvement of educational level. According to the above analysis, it can be found that with the improvement of education level, the intermediary effect decreases; that is, the mediation effect is regulated by education level. Therefore, hypothesis H3 is verified.

Next, income, age, gender, and marriage were taken as covariates to test the regulatory role of BMI in the intermediary process of image construction affecting food waste behavior through emotion. The results are shown in Table A4 (in Appendix A). The results of data analysis show that BMI can adjust the mediation model. When the mediating variable is negative emotion and the BMI is negative by one standard deviation, the 95% bootstrap confidence interval contains 0, and the mediating effect is not significant; however, BMI has a significant regulatory effect in other cases. When the BMI is different, the size of the mediation effect will also be different; that is, when BMI is high, the positive impact of image construction on individual food waste behavior through emotion is more significant; with the improvement of BMI level, such positive impact increases gradually. According to the above analysis, it can be found that with the improvement of BMI, the mediating effect increases; that is, the mediation effect is regulated by BMI. Therefore, hypothesis H4 is verified.

Furthermore, this study explored the regulatory effect of education level and BMI on the direct prediction of food waste behavior by image construction demand. The results are shown in Table A5 and Table A6 (in Appendix A). We observed that after putting the education level into the model, the product of appearance image construction and education level can significantly predict the exclusion, disregard, and extravagant type waste behavior, and the product of social image construction and education level can significantly predict the four types of waste behavior. After putting BMI into the model, the product of the appearance image construction and BMI only has a significant predictive effect on the exclusion waste behavior; and the product of the social image construction and BMI has a significant predictive effect on the four types of waste behavior.

In terms of the moderating effect, the positive relationship between image construction and wasteful behavior gradually weakens as the level of education increases from low to high. This indicates that education level has a negative moderating effect in the process of image construction influencing wasteful behavior. As BMI goes from low to high, the positive correlation between image construction and wasteful behavior gradually strengthens, indicating that BMI has a positive regulatory role in the process of image construction affecting wasteful behavior. Therefore, hypotheses H5 and H6 are verified.

**Figure 2:**
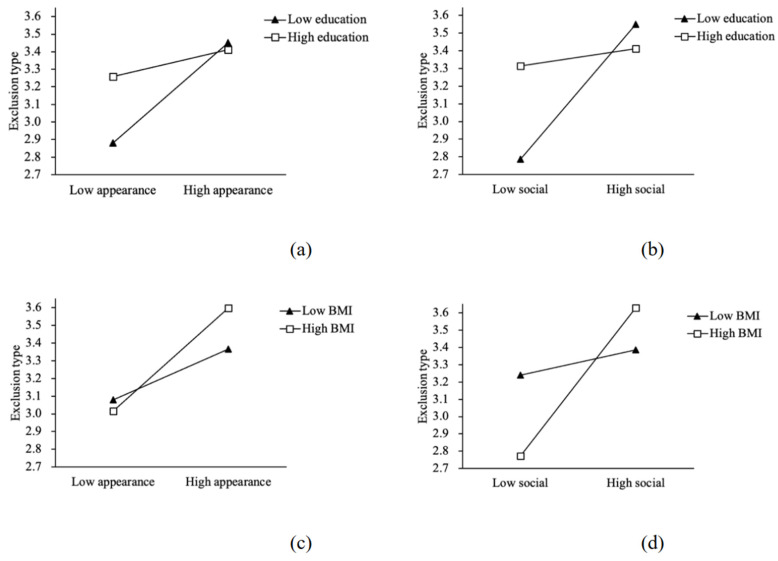
Diagram of the moderating effect. Note: (**a**,**b**) describe the moderating effect of education level on image construction to predict exclusion waste behavior; (**c**,**d**) describe the moderating effect of body mass index on image construction to predict exclusion waste behavior.

Figure 2 draws a diagram of the moderating effect by selecting appearance image and social image as independent variables and exclusion waste as dependent variables. The graph visualizes the moderating effect of education level and BMI on the predicted pathway from image construction needs to individual food waste behavior.

From Figure 2a,b, it can be seen that with the improvement of individual education level, the predictive effect of image construction on exclusion waste behavior gradually decreases, indicating that education level plays a negative role in regulating. As shown in Figure 2c,d, the predictive effect of image construction on exclusion wasteful behavior tends to increase gradually as BMI increases, indicating that BMI plays a positive moderating role.

## 5. Discussion

This research constructs a model of the formation mechanism of individual food waste behavior from the perspective of image construction, and expands the research content of the driving factors of individual food waste behavior from the motivation of appearance image construction and social image construction. According to the research results, the needs of appearance image construction and social image construction stimulate the formation and aggravation of food waste behavior. These findings show that individuals who focus on image construction do not care about the possible negative consequences of food waste behavior. Individuals with high demand for appearance image construction pay more attention to appearance and external evaluation, and tend to pursue a perfect body [11]. These individuals will pay more attention to the perceived benefits brought by food waste behavior in the process of eating; thus, the possibility of food waste behavior is higher. Moreover, individuals with high demand for social image construction are more eager for social identity than other individuals [13]. Piras et al. [62] conducted a study in Italy and found that individuals may gain a sense of social recognition through food waste behavior. In this case, it will make these individuals ignore the negative results of food waste behavior and tend to be extravagant and wasteful in social interactions. The result indicates that in the intervention process of guiding individuals to reduce food waste behavior, changing the wrong demand for paying too much attention to appearance is as important as improving the bad social atmosphere.

In addition, emotional fluctuations positively mediate the transformation from image construction needs to food waste behavior, which is similar to the conclusion of Tsaur [63] The researches of Portnoy [64] and Xu [65] have emphasized the conduction role of emotion in the path from external stimuli to behavioral results. Folkman [66] found that positive emotions and negative emotions are not completely opposed. External demand stimuli may cause these two emotions to occur simultaneously, and emotions are the main factor that triggers behavior motivation [67]. Russell et al. [68] selected 172 British residents for a 14-month follow-up survey and found that emotion plays an important role in driving food waste behavior. Based on stimulus-organism-response (S-O-R) theory, the mental processes that lie between external demand stimuli and behavioral responses are closely related to the behavioral responses made by individuals [69]. That is, the need for image construction will stimulate emotions to fluctuate. Whether the direction of emotional fluctuations is positive or negative, it will drive the intensification of individual food waste behavior.

The results of the interaction effect analysis show that with the improvement of individual education level, the predictive effect of image construction on exclusion waste behavior gradually decreases. With the increase of BMI, the predictive effect of image construction on exclusion waste behavior gradually increases. The knowledge-attitude-practice model demonstrates that practice is related to knowledge and attitudes [70]; individuals with high education level have greater environmental knowledge and awareness of conservation and thus tend to show low levels of food waste behavior in practice. This result is also confirmed by the research of Abeliotis [71]. The individual’s desire for appearance image is a blind pursuit attitude, which may have a positive impact on the individual’s intention to follow his/her food waste behavior to maintain his/her figure. This indicates that when the individual’s education reaches a certain level, it will buffer the transformation from the needs for image construction to food waste behavior. On the contrary, when the individual BMI reaches a certain critical value, it will play an excessive role in promoting the transformation from the need for image construction to food waste behavior.

The findings of this research in the field of food waste behavior will further enrich and refine the theoretical research on the psychological mechanism and formation conditions behind the waste behavior. Notably, food waste behavior has nothing to do with the direction of emotional fluctuation, but only with whether there is emotional fluctuation. This is similar to the conclusion of Annesi [72]. Annesi [72] pointed out that emotional fluctuations can trigger behavioral problems, and the degree of food waste behavior is related to the amplitude of emotional fluctuations. The results of our study indicate that different levels of education are related to significant differences in food waste behavior. The finding may be because the level of education is generally linked to individuals’ environmental attitudes [73]. However, Pocol et al. [24] carried out a study in Romania and found that education level is not always a positive predictor of food waste behavior, which may be related to cultural differences across countries. Jinhee [19] pointed out that individuals with high BMI are less sensitive to resource conservation and are less concerned about the negative consequences of food waste. Our study further explores this conclusion. As BMI goes from low to high, the positive correlation between image construction and wasteful behavior gradually increases. This study attempts to ensure the reliability of the sample data as much as possible during the research process, but it may have some limitations because it is difficult to completely avoid the disturbance of the subjects from the epidemic situation. In general, this study introduces the image construction factors into the research of food waste behavior and constructs the formation mechanism model of individual food waste behavior, hoping to enrich and expand the research in the related fields of food waste behavior. Moreover, this study helps to provide a theoretical basis for the government to formulate policies to reduce food waste behavior. The government can fundamentally improve the current situation of food waste and further promote the sustainable development of society by properly intervening and guiding individual food waste behavior along a scientific path.

## 6. Conclusions and Recommendation

### 6.1. Research Conclusions

Effectively intervening in individuals to reduce food waste behavior at the source of waste (i.e., guiding individual rational consumption and resource conservation awareness) is a fundamental measure to reduce the rate of food waste. From the perspective of individual behavior motivation, this study discusses the mechanism of emotion, education level, and BMI in the transmission process from image construction to food waste behavior. Data from 749 subjects showed that the need for image construction is significantly correlated with individual food waste behavior. Moreover, the hierarchical regression results show that positive emotions and negative emotions partially mediate the promotion of image construction on food waste behavior. Specifically, for exclusion and interference type wasteful behavior, the mediating effect of positive emotions is stronger; for disregard and extravagant type wasteful behavior, the mediating effect of negative emotions is stronger. Individual heterogeneity interferes with individual behavior. With the improvement of individual education level, the predictive effect of image construction gradually decreases; that is, education level negatively regulates food waste behavior. In contrast, BMI positively regulates food waste behavior. The research results provide important theoretical support and practical reference significance for reducing food waste behavior.

### 6.2. Policy Suggestions

In order to promote sustainable social development, China has implemented the “clear your plate” campaign since 2013. At the end of 2013, China enacted the “Regulations on Party and Government Organs to Exercise Rigorous Economy and Oppose Waste” to spearhead an anti-food waste policy among government workers. In 2021, the central government enacted “The Law of the People’s Republic of China Against Food Waste”, implying that the public will face penalties for food waste behavior. It is undeniable that these policies have achieved certain results. However, reducing food waste behavior is a complex and long-term process. The government needs to constantly improve policies and combine a variety of measures to reduce food waste. Therefore, based on the results of this research, the following policy recommendations are put forward to encourage individuals to reduce food waste and promote the sustainable development of resources.

(1) Individual needs should be guided across levels. The study found that individuals with high demand for image construction are not sensitive to the negative results caused by food waste, and lack the psychological tendency to pay attention to the ecological environment and save food. These individuals believe that the social benefits gained from image construction can offset the serious consequences of food waste, and thus they are not willing to reduce wasteful behavior for the sake of a good ecological environment. Therefore, government should reduce this wrong demand through behavioral intervention and induce demand conducive to individual positive behavior. For example, negative results such as serious waste of resources can stimulate individuals’ obligations and sense of responsibility for food and the environment, and stimulate the generation of self-realization needs. In the intervention measures that guide the right direction of demand, policy makers can actively guide individuals to form social master needs, or encourage individuals’ desires and longing for a green environment and an atmosphere of diligence and thrift. Through various forms of communication, individuals are encouraged to reduce their attention to the needs of image construction and improve their sensitivity to the adverse consequences of food waste behavior. In this way, individuals will eventually meet their self-realization needs due to the reduction of food waste.

(2) Diversified emotional regulation methods can be adopted. The results show that individuals may be affected by emotional fluctuations that significantly increase the probability of food waste behavior. In reality, individuals gain social recognition in the process of adapting to the social environment, participating in social life, learning social norms, and performing social roles, which reflects their characteristics and needs in terms of emotions. Therefore, individuals tend to be manipulated by emotions and have emotional fluctuations. When individuals have emotional fluctuations, they will produce special physiological behavior to regulate emotions and heal psychological wounds. Eating is a daily behavior, and most people will adjust their emotions by changing the amount of food they eat, which will lead to serious food waste behavior. When individuals have emotional fluctuations, they should avoid regulating their emotions through food waste, and shift their attention to other directions (e.g., chatting with others, meditation, and thinking), or actively change the environment (e.g., going out for a walk, traveling, and visiting).

(3) Environmental knowledge education should be emphasized and differentiated intervention should be implemented. The results of this study show that education level has a negative regulating effect in the process of image construction affecting wasteful behavior, and BMI has a positive regulating effect in the process of image construction affecting wasteful behavior. Years of school education have had a subtle impact on individuals. Individuals with high education levels have a higher awareness of environmental protection and resource conservation. From the perspective of education level, exploring measures to reduce food waste can provide some valuable information. In terms of specific operation, government should constantly expand high-quality educational resources to make the scale, layout, structure, and quality of education meet the needs of society. Moreover, schools can use contextual intervention during education to incorporate environmental knowledge into teaching, and increase students’ understanding of the hazards of food waste and enhance knowledge learning and individual independent thinking ability through classroom learning. In this way, it is possible to root the awareness of caring for the environment and saving resources in students’ instincts, and thus fundamentally reduce individual food waste behavior. Furthermore, the government should launch a nationwide campaign with the theme of saving food and eliminating waste, so that environmental knowledge education can cover all groups and further guide the majority of individuals to establish the concept of saving glory and develop the habit of diligence and thrift. Additionally, in view of the differences in individual BMI, government can take targeted diversified intervention measures to gradually reduce the rate of food waste.

## Figures and Tables

**Table 1:** Demographic characteristics of the sample.

Variable		Frequency	Variable		Frequency
Gender	Male	38.9%	Education background	High school or below	28.0%
	Female	61.1%		Junior college	8.4%
Marital status	Married	43.9%		Bachelor	53.9%
	Unmarried	56.1%		Master	8.7%
Age	≤17	1.1%		Doctor	0.9%
	18–25	47.5%	Number of household members	1	2.1%
	26–30	8.3%		2	7.9%
	31–40	11.2%		3	37.4%
	41–50	17.8%		4	29.1%
	51–60	10.3%		≥5	23.5%
	≥61	3.9%	Monthly income	<3000	18.7%
Body mass index	Too light (<18)	15.6%		3000–5000	30.3%
	Normal (18–23.9)	59.9%		5000–10,000	36.8%
	Overweight (24–27)	19.9%		10,000–20,000	10.7%
	Obese (>27)	4.5%		>20,000	3.5%

**Table 2:** Factor loading matrix after orthogonal rotation of the dependent variable.

Item	Components			
	1	2	3	4
Exclusion 2	0.884			
Exclusion 1	0.867			
Exclusion 3	0.772			
Extravagant 1		0.824		
Extravagant 3		0.721		
Extravagant 2		0.689		
Interference 1			0.813	
Interference 2			0.716	
Disregard 2				0.793
Disregard 1				0.715

**Table 3:** Descriptive statistics and correlation coefficients.

	External Image	Social Image	Positiveemotion	Negative Emotion	Wasteful Behavior	Education	Body Mass Index
External image	1						
Social image	0.256 ***	1					
Positive emotion	0.308 ***	0.387 ***	1				
Negative emotion	0.176 ***	0.239 ***	0.363 ***	1			
Wasteful behavior	0.242 ***	0.31 ***	0.444 ***	0.641 ***	1		
Education	0.045	0.015	0.154 ***	−0.026	0.057	1	
Body mass index	−0.032	0.036	−0.025	−0.005	−0.01	−0.16 ***	1

Note: *n* = 749, *** means *p* < 0.001; the data on the diagonal is the internal consistency coefficient of each variable.

**Table 4:** Predictive effect of image construction on food waste behavior.

	Exclusion Type	Disregard Type	Extravagant Type	Interference Type
Constant	0.861 *	1.668 ***	1.020 *	0.772 *
Income	0.070	0.058	0.048	0.026
Age	−0.030	−0.057	−0.015	−0.005
Gender	0.133	−0.047	−0.164 *	−0.129
Marriage	0.400 **	−0.109	−0.002	0.192
R^2^	0.052	0.008	0.014	0.010
F	10.157 ***	1.481	2.580 *	1.810
Appearance image	0.164 ***	0.139 ***	0.149 ***	0.175 ***
Social image	0.251 ***	0.220 ***	0.330 ***	0.315 ***
R^2^	0.137	0.062	0.118	0.111
F	19.705 ***	8.210 ***	16.594 ***	15.417 ***

Note: *n* = 749, * means *p* < 0.05, ** means *p* < 0.01, *** means *p* < 0.001.

## Data Availability

The data presented in this study are available on request from author.

## Appendix A

**Table A1:** Analysis of mediating effects based on appearance image.

	β	SE	CI	β	SE	CI	β	SE	CI	β	SE	CI
	X1→M1→Y1			X1→M1→Y2			X1→M1→Y3			X1→M1→Y4		
Total Effect	0.220	0.036	(0.149, 0.291)	0.187	0.040	(0.108, 0.267)	0.222	0.039	(0.146, 0.298)	0.245	0.040	(0.166, 0.324)
Direct Effect	0.139	0.033	(0.075, 0.204)	0.072	0.034	(0.006, 0.138)	0.106	0.032	(0.044, 0.168)	0.143	0.035	(0.074, 0.212)
Indirect Effect	0.080	0.020	(0.043, 0.121)	0.116	0.027	(0.065, 0.171)	0.116	0.027	(0.064, 0.170)	0.102	0.025	(0.056, 0.153)
	X1→M2→Y1			X1→M2→Y2			X1→M2→Y3			X1→M2→Y4		
Total Effect	0.220	0.036	(0.149, 0.291)	0.187	0.040	(0.108, 0.267)	0.222	0.039	(0.146, 0.298)	0.245	0.040	(0.166, 0.324)
Direct Effect	0.094	0.035	(0.026, 0.163)	0.084	0.041	(0.004, 0.163)	0.114	0.039	(0.039, 0.190)	0.129	0.040	(0.051, 0.207)
Indirect Effect	0.126	0.021	(0.087, 0.171)	0.104	0.019	(0.068, 0.143)	0.108	0.019	(0.072, 0.145)	0.116	0.019	(0.080, 0.156)

**Table A2:** Analysis of the mediating effect based on social image.

	β	SE	CI	β	SE	CI	β	SE	CI	β	SE	CI
	X2→M1→Y1			X2→M1→Y2			X2→M1→Y3			X2→M1→Y4		
Total Effect	0.301	0.042	(0.219, 0.383)	0.262	0.047	(0.170, 0.354)	0.375	0.044	(0.288, 0.462)	0.368	0.046	(0.278, 0.458)
Direct Effect	0.181	0.039	(0.105, 0.257)	0.085	0.040	(0.007, 0.164)	0.203	0.037	(0.130, 0.276)	0.216	0.041	(0.135, 0.297)
Indirect Effect	0.120	0.025	(0.074, 0.170)	0.177	0.035	(0.109, 0.250)	0.172	0.034	(0.107, 0.241)	0.152	0.031	(0.095, 0.216)
	X2→M2→Y1			X2→M2→Y2			X2→M2→Y3			X2→M2→Y4		
Total Effect	0.301	0.042	(0.219, 0.383)	0.262	0.047	(0.170, 0.354)	0.375	0.044	(0.288, 0.462)	0.368	0.046	(0.278, 0.458)
Direct Effect	0.114	0.042	(0.031, 0.197)	0.108	0.049	(0.011, 0.205)	0.231	0.047	(0.140, 0.323)	0.206	0.048	(0.112, 0.300)
Indirect Effect	0.187	0.029	(0.132, 0.246)	0.154	0.027	(0.103, 0.207)	0.144	0.025	(0.097, 0.197)	0.162	0.027	(0.113, 0.218)

**Table A3:** The mediating effect of emotion at different levels of education.

Negative emotion	**Edu**	**β**	**SE**	**CI**	**β**	**SE**	**CI**	**β**	**SE**	**CI**	**β**	**SE**	**CI**
	Path	X1→M1→Y1			X1→M1→Y2			X1→M1→Y3			X1→M1→Y4		
	M-SD	0.139	0.026	(0.089, 0.189)	0.200	0.036	(0.131, 0.273)	0.202	0.037	(0.131, 0.147)	0.181	0.034	(0.116, 0.248)
	M	0.064	0.018	(0.029, 0.101)	0.093	0.026	(0.040, 0.144)	0.094	0.027	(0.042, 0.147)	0.084	0.230	(0.037, 0.131)
	M + SD	−0.010	0.025	(−0.059, 0.040)	−0.015	0.035	(−0.083, 0.055)	−0.015	0.036	(−0.087, 0.056)	−0.013	0.032	(−0.077, 0.048)
	Path	X2→M1→Y1			X2→M1→Y2			X2→M1→Y3			X2→M1→Y4		
	M-SD	0.169	0.032	(0.108, 0.233)	0.250	0.044	(0.164, 0.338)	0.247	0.044	(0.160, 0.336)	0.217	0.042	(0.139, 0.301)
	M	0.099	0.022	(0.057, 0.145)	0.146	0.033	(0.084, 0.212)	0.144	0.032	(0.085, 0.209)	0.127	0.029	(0.072, 0.188)
	M + SD	0.028	0.031	(−0.034, 0.089)	0.042	0.046	(−0.047, 0.135)	0.041	0.045	(−0.046, 0.133)	0.036	0.041	(−0.042, 0.117)
Positive emotion	**Edu**	**β**	**SE**	**CI**	**β**	**SE**	**CI**	**β**	**SE**	**CI**	**β**	**SE**	**CI**
	Path	X1→M2→Y1			X1→M2→Y2			X1→M2→Y3			X1→M2→Y4		
	M-SD	0.125	0.027	(0.074, 0.180)	0.107	0.024	(0.061, 0.157)	0.111	0.024	(0.064, 0.160)	0.118	0.026	(0.070, 0.169)
	M	0.124	0.020	(0.085, 0.165)	0.105	0.019	(0.070, 0.145)	0.109	0.019	(0.074, 0.146)	0.116	0.019	(0.080, 0.156)
	M + SD	0.122	0.025	(0.076, 0.174)	0.104	0.022	(0.064, 0.150)	0.108	0.023	(0.067, 0.155)	0.115	0.024	(0.071, 0.164)
	Path	X2→M2→Y1			X2→M2→Y2			X2→M2→Y3			X2→M2→Y4		
	M-SD	0.214	0.036	(0.146, 0.285)	0.178	0.032	(0.116, 0.240)	0.170	0.031	(0.114, 0.233)	0.191	0.033	(0.128, 0.259)
	M	0.164	0.027	(0.112, 0.217)	0.136	0.024	(0.090, 0.184)	0.130	0.023	(0.088, 0.178)	0.146	0.025	(0.099, 0.197)
	M + SD	0.114	0.029	(0.058, 0.172)	0.094	0.026	(0.046, 0.147)	0.090	0.025	(0.045, 0.142)	0.101	0.027	(0.052, 0.156)

**Table A4:** The mediating effect of emotion at different levels of body mass index (BMI).

Negative emotion	**BMI**	**β**	**SE**	**CI**	**β**	**SE**	**CI**	**β**	**SE**	**CI**	**β**	**SE**	**CI**
	Path	X1→M1→Y1			X1→M1→Y2			X1→M1→Y3			X1→M1→Y4		
	M-SD	0.045	0.034	(−0.034, 0.100)	0.065	0.049	(−0.049, 0.143)	0.065	0.049	(−0.051, 0.143)	0.058	0.044	(−0.046, 0.128)
	M	0.078	0.019	(0.042, 0.118)	0.113	0.027	(0.062, 0.170)	0.114	0.027	(0.063, 0.169)	0.101	0.025	(0.055, 0.152)
	M + SD	0.112	0.037	(0.056, 0.202)	0.162	0.054	(0.083, 0.295)	0.162	0.055	(0.081, 0.296)	0.144	0.050	(0.070, 0.265)
	Path	X2→M1→Y1			X2→M1→Y2			X2→M1→Y3			X2→M1→Y4		
	M-SD	0.043	0.038	(−0.039, 0.108)	0.063	0.056	(−0.059, 0.166)	0.062	0.055	(−0.055, 0.158)	0.055	0.049	(−0.049, 0.143)
	M	0.113	0.024	(0.068, 0.161)	0.168	0.035	(0.101, 0.237)	0.164	0.033	(0.101, 0.230)	0.145	0.030	(0.087, 0.208)
	M + SD	0.183	0.043	(0.108, 0.277)	0.272	0.061	(0.162, 0.399)	0.265	0.060	(0.158, 0.391)	0.235	0.055	(0.142, 0.354)
Positive emotion	**BMI**	**β**	**SE**	**CI**	**β**	**SE**	**CI**	**β**	**SE**	**CI**	**β**	**SE**	**CI**
	Path	X1→M2→Y1			X1→M2→Y2			X1→M2→Y3			X1→M2→Y4		
	M-SD	0.098	0.026	(0.047, 0.151)	0.081	0.022	(0.039, 0.125)	0.084	0.022	(0.041, 0.130)	0.091	0.024	(0.043, 0.139)
	M	0.124	0.021	(0.085, 0.167)	0.103	0.019	(0.068, 0.141)	0.106	0.018	(0.072, 0.144)	0.115	0.019	(0.079, 0.156)
	M + SD	0.150	0.031	(0.090, 0.214)	0.124	0.027	(0.075, 0.181)	0.129	0.027	(0.078, 0.184)	0.139	0.029	(0.085, 0.199)
	Path	X2→M2→Y1			X2→M2→Y2			X2→M2→Y3			X2→M2→Y4		
	M-SD	0.104	0.035	(0.032, 0.168)	0.085	0.029	(0.028, 0.140)	0.079	0.027	(0.024, 0.133)	0.091	0.030	(0.027, 0.145)
	M	0.177	0.028	(0.124, 0.235)	0.144	0.025	(0.097, 0.195)	0.135	0.024	(0.090, 0.185)	0.154	0.025	(0.105, 0.203)
	M + SD	0.249	0.045	(0.169, 0.346)	0.204	0.041	(0.130, 0.289)	0.191	0.038	(0.122, 0.273)	0.218	0.041	(0.142, 0.303)

**Table A5:** Direct effects at different levels of education.

	Edu	β	SE	CI	β	SE	CI	β	SE	CI	β	SE	CI
Appearance image	Path	Exclusion			Disregard			Extravagant			Interference		
	M-SD	0.286	0.045	(0.197, 0.374)	0.259	0.051	(0.160, 0.359)	0.275	0.049	(0.179, 0.370)	*p* = 0.475 (−0.103, 0.048)		
	M	0.200	0.037	(0.127, 0.272)	0.168	0.041	(0.087, 0.249)	0.208	0.040	(0.130, 0.286)			
	M + SD	0.113	0.056	(0.003, 0.224)	0.077	0.063	(−0.047, 0.200)	0.142	0.060	(0.023, 0.261)			
Social image	Path	Exclusion			Disregard			Extravagant			Interference		
	M-SD	0.443	0.051	(0.344, 0.543)	0.412	0.057	(0.300, 0.524)	0.480	0.054	(0.374, 0.587)	0.479	0.056	(0.368, 0.590)
	M	0.247	0.042	(0.163, 0.330)	0.209	0.048	(0.115, 0.303)	0.339	0.045	(0.250, 0.428)	0.329	0.047	(0.236, 0.422)
	M + SD	0.050	0.066	(−0.079, 0.179)	0.007	0.074	(−0.139, 0.152)	0.198	0.070	(0.060, 0.336)	0.179	0.073	(0.036, 0.323)

**Table A6:** Direct effects at different levels of body mass index (BMI).

	BMI	β	SE	CI	β	SE	CI	β	SE	CI	β	SE	CI
Appearance image	Path	Exclusion			Disregard			Extravagant			Interference		
	M-SD	0.135	0.057	(0.022, 0.247)	*p* = 0.334 (−0.009, 0.028)			*p* = 0.317 (−0.009, 0.027)			*p* = 0.879 (−0.020, 0.017)		
	M	0.216	0.036	(0.146, 0.287)									
	M + SD	0.298	0.054	(0.191, 0.405)									
Socia image	Path	Exclusion			Disregard			Extravagant			Interference		
	M-SD	0.081	0.075	(−0.065, 0.228)	0.068	0.084	(−0.097, 0.232)	0.196	0.079	(0.041, 0.352)	0.220	0.083	(0.058, 0.382)
	M	0.286	0.042	(0.204, 0.368)	0.249	0.047	(0.157, 0.341)	0.363	0.044	(0.276, 0.450)	0.358	0.046	(0.267, 0.449)
	M + SD	0.490	0.068	(0.357, 0.623)	0.430	0.076	(0.280, 0.580)	0.529	0.072	(0.388, 0.670)	0.496	0.075	(0.348, 0.643)
